# Insights on the Association between Thyroid Diseases and Colorectal Cancer

**DOI:** 10.3390/jcm12062234

**Published:** 2023-03-13

**Authors:** Federica Gagliardi, Enke Baldini, Eleonora Lori, Silvia Cardarelli, Daniele Pironi, Augusto Lauro, Domenico Tripodi, Piergaspare Palumbo, Eleonora D’Armiento, Giuseppe Cavallaro, Andrea Polistena, Valerio D’Orazi, Simone Sibio, Poupak Fallahi, Alessandro Antonelli, Vito D’Andrea, Salvatore Ulisse, Salvatore Sorrenti

**Affiliations:** 1 Department of Surgery, “Sapienza” University of Rome, 00161 Rome, Italy; 2 Department of Internal Medicine and Medical Specialties, “Sapienza” University of Rome, 00161 Rome, Italy; 3 Department of Translational Research and New Technologies in Medicine and Surgery, University of Pisa, 56126 Pisa, Italy; 4 Department of Surgical, Medical and Molecular Pathology and of Critical Area, University of Pisa, 56126 Pisa, Italy

**Keywords:** thyroid, hypothyroidism, hyperthyroidism, colorectal cancer

## Abstract

Benign and malignant thyroid diseases (TDs) have been associated with the occurrence of extrathyroidal malignancies (EMs), including colorectal cancers (CRCs). Such associations have generated a major interest, as their characterization may provide useful clues regarding diseases’ etiology and/or progression, with the possible identification of shared congenital and environmental elements. On the other hand, elucidation of the underlying molecular mechanism(s) could lead to an improved and tailored clinical management of these patients and stimulate an increased surveillance of TD patients at higher threat of developing EMs. Here, we will examine the epidemiological, clinical, and molecular findings connecting TD and CRC, with the aim to identify possible molecular mechanism(s) responsible for such diseases’ relationship.

## 1. Introduction

Thyroid diseases (TDs) have been associated with the occurrence of a number of extra-thyroidal malignancies (EM), including colorectal cancers (CRCs) [1,2,3,4,5,6,7,8,9,10,11,12,13,14,15,16,17,18]. These associations have generated a great interest based on the opportunity to detect possible shared congenital and environmental elements liable for diseases’ etiology and/or progression. In a clinical setting, a comprehensive definition of the pathogenic events underlying these associations could foster an increased surveillance of TD patients at higher threat of developing EMs and allow for an improved and tailored clinical management of patients. This review begins with a brief general description of TD and CRC, after which epidemiological, clinical, and experimental findings connecting TD and colorectal cancer (CRC) are discussed, and possible molecular mechanism(s) responsible for such a relationship are considered. To this end, we examined articles present in the PubMed database by entering the following keywords in the advanced search builder: colorectal cancer; thyroid disease; colorectal cancer AND thyroid disease/hypothyroidism/hyperthyroidism/thyroid cancer/thyroid hormones/TSH/TRH. Peer reviewed, original research articles in English published until November 2022 were included.

### 1.1. Colorectal Cancer: An Overview

Colorectal cancer (CRC) is the third most common malignancy and the second deadliest cancer worldwide. The American Cancer Society **estimated** 106,180 new cases of colon cancer and 44,850 new cases of rectal cancer in the United States for 2022 [19]. The risk of CRC increases with age: 90% of all diagnoses concern patients over 50, with an average age of 68 and 72 years for men and women, respectively [20]. CRC incidence rate is 30% higher in men than in women, while the lifetime risk of developing CRC is similar in both sexes [21]. The reason why the gender affects CRC onset is not yet fully understood, but it could be related to differences in the exposure to risk factors such as cigarette smoking, alcohol consumption, and dietary patterns. CRC appears to be positively correlated with the Human Development Index (HDI), in that the most significant increases in CRC incidence and mortality occur in medium and high HDI countries [22]. Overweight and obesity, diet high in red and processed meat and low in fiber, fruits, and vegetables, physical inactivity, smoking, and alcohol are well-known modifiable risk factors for colorectal neoplasms [23,24,25,26,27].

CRC is paradigmatic of the carcinogenesis model defined as “multistep”, in which the adenoma–carcinoma transformation progresses owing to the stepwise accumulation of well-characterized genetic and epigenetic alterations, possibly caused by the above-mentioned factors [28,29]. Among these, activating mutations of components of the WNT/β-Catenin pathway are frequently encountered [30,31,32,33,34,35]. Approximately 60–65% of all CRCs are sporadic cases, while in approximately 25% of patients, a family history of CRC is present, with a first-degree relative showing two to four times higher risk of developing CRC compared to people without family history [28,29]. Furthermore, in approximately 5% of cases, CRC may be part of the clinical presentation of a hereditary cancer syndrome [36]. Among the latter, the two most common are autosomal dominant disorders: the hereditary nonpolyposis CRC or Lynch syndrome, caused by inactivating mutations in one of four genes belonging to the mismatch repair system, namely MSH2, MLH1, MSH6, and PMS2 [37], and the familial adenomatous polyposis (FAP), due to germline mutations in the tumor suppressor gene adenomatous polyposis coli (APC) [38]. The syndrome is characterized by the development of hundreds or thousands of adenomas in the colorectum and, if untreated, it associates with a 100% lifetime risk of malignant transformation [38]. It is worthwhile to mention that, irrespective of whether CRCs arise sporadically or in patients with a family history of CRC, environmental factors may play a prominent role in CRC pathogenesis. This is strongly suggested by the observation that a considerable number of CRCs occurring in individuals with a positive family history are not inherited, as they bear de novo acquired genomic alterations [28,39].

The vast majority of CRCs (approximately 90%) are adenocarcinomas originated from cells of the colorectal mucosal epithelium [28]. Uncommon variants, accounting for less than 10% of CRCs, are adenosquamous, neuroendocrine, squamous cell, and undifferentiated carcinomas [40]. Treatment of CRC involves a multidisciplinary approach comprising surgery, chemotherapy, and radiation therapy to reduce local recurrence rates and improve survival outcomes.

### 1.2. Thyroid Diseases: An Overview

The expression “thyroid diseases” (TDs) refers to a wide and heterogeneous group of clinical pathological conditions. According to the American Thyroid Association, the lifetime risk to develop a TD is approximately 12%, and it is five to eight times higher in women than in men [41]. In iodine-depleted areas, TD are largely represented by hypothyroidism and diffuse goiter, which can develop into nodular goiter, while autoimmune TD (AITD), namely Hashimoto thyroiditis (HT) leading to hypothyroidism, and Graves’ disease (GD) causing hyperthyroidism, are prevalent in iodine-replete areas [41].

Hypothyroid patients are characterized by fatigue, cold intolerance, weight gain, bradycardia, constipation, hair loss/thinning, dry skin, muscle aches, and reversible cognitive impairment [42]. Besides iodine deficiency and the chronic autoimmune Hashimoto thyroiditis mentioned above, the causes for primary hypothyroidism include congenital hypothyroidism, due to either thyroid dysgenesis or dyshormonogenesis, use of drugs (e.g., amiodarone, lithium, thalidomide, interferon-α, tyrosine kinase inhibitors, etc.), iatrogenic causes such as thyroidectomy, and/or radioiodine treatment [42]. Secondary or central hypothyroidism may arise from pituitary or hypothalamic dysfunctions, or resistance to TRH (thyrotropin-releasing hormone) or TSH (thyrotropin). Finally, inactivating mutations of the thyroid hormone (TH) receptor genes THRA and THRB or the TH transmembrane transporter gene MCT8 result in peripheral hypothyroidism due to a reduced sensitivity of target tissues to TH [42].

Common features of hyperthyroidism include weight loss, tachycardia, tremors, anxiety, irritability, heat intolerance, intestinal hypermotility, decreased cognitive function, and depression [42]. Hyperthyroidism occurs following an excess of TH biosynthesis and release or following thyroxine overtreatment in patients affected by hypothyroidism, non-toxic nodular goiter, or thyroid cancer [42]. Regarding the former, the main cause is the above-mentioned autoimmune Graves’ disease, characterized by the presence of stimulating TSH receptor antibodies. Other causes comprise toxic adenoma or multinodular goiter, thyroiditis inducing release of TH from the storage compartments of the inflamed thyroid gland, drugs such as amiodarone, interferon-α, lithium, tyrosine kinase inhibitors, antiretroviral therapies, immune checkpoint mediators, etc. [42].

Thyroid nodules are frequently encountered in clinical practice. The vast majority of them are benign, but a minority (7–15%) conceals a malignant lesion [43,44]. Differentiated thyroid carcinomas (DTCs) represent 90–95% of newly diagnosed thyroid cancers, and include the papillary (PTC) and follicular (FTC) histotypes, accounting for 85% and 10% of cases, respectively [43,44,45]. Approximately 10–15% of DTCs will progress into dedifferentiated and more aggressive variants, i.e., the poorly differentiated thyroid carcinomas (PDTCs) and the incurable anaplastic thyroid carcinomas (ATCs) [45]. Exposure to ionizing radiation, chromosomal and genetic alterations, iodine deficiency, TSH level, AITD, gender, estrogens, obesity, lifestyle changes, and environmental pollutants are some possible risk factors [45,46]. In recent years, the comprehension of the molecular mechanisms underlying thyroid cancer progression has considerably improved with the identification of oncogenic drivers in more than 98% of cases, leading to a new molecular classification of tumors that could ameliorate patients’ clinical management [47,48]. The utmost genetic alterations encountered in DTC are represented by activating mutations of genes implicated in the mitogen-activated protein kinase (MAPK) pathway (i.e., BRAF and RAS genes), or fusions of the RET and NTRK1 genes [47,48]. The evolution from DTC to PDTC and ATC depends on the occurrence of further mutations, as those of the p53 and the telomerase reverse transcriptase (TERT) genes, and by induction in malignant cells of the epithelial–mesenchymal transition [49,50].

Surgery and radioiodine therapy followed by TSH suppression therapy is the treatment of choice in patients affected by DTC [43,51]. The most significant prognostic factor is represented by the histotype: the survival rate at 5 years is approximately 99.7% for PTC, 97.3% for FTC, and less than 20% at 1 year for ATC [52,53]. For PDTC and ATC, a number of clinical trials with novel targeted therapies are currently under evaluation [54,55,56,57].

## 2. Epidemiological Evidence on Thyroid Dysfunctions and Colorectal Cancer

### 2.1. Hypothyroidism and Colorectal Cancer

The first epidemiological evidence suggesting a role of hypothyroidism in the pathogenesis and progression of CRC came from Rennert and colleagues in 2010, who performed a population-based matched case–control study in northern Israel on incident CRC patients and control subjects (2566 pairs) under levothyroxine treatment for at least 5 years (Table 1) [58]. These authors reported that the use of levothyroxine was associated with a significant reduction in the CRC odds ratio (OR = 0.59). This association remained statistically significant after adjustment for age, gender, use of aspirin and statins, sports activity, family history of CRC, ethnic group, and level of vegetable consumption [58]. In a similar study performed in northern California on a larger cohort, including 4729 rectum/rectosigmoid cancer patients and 12,207 colon cancer patients, Friedman and colleagues reported that the risk of rectal cancer was more than 30% lower in men who used levothyroxine for at least 5 years, relative to the reference group [59]. A slight reduction in risk also appeared for colon cancer, although not statistically significant (Table 1). Likewise, the cancer risk at both sites was diminished in women, even if the statistical significance was borderline [59]. Later on, Chen and colleagues, by analyzing 1521 HT patients and 6084 frequency-matched non-HT patients in Taiwan, showed that the HT cohort had a hazard ratio (HR) of 4.76 for developing CRC compared with a non-HT cohort [5]. Similar observations were reported in China by Mu and colleagues, who performed a case–control study including 273 CRC patients and 819 controls [60]. Their results indicated that the prevalence of subclinical hypothyroidism was significantly higher in patients affected by CRC (24.5%) than in controls (14.3%) [60]. By dividing the entire study population into euthyroid and subclinical hypothyroid patients, they found a significantly higher prevalence of CRC in subclinical hypothyroid patients (34.9%) compared to euthyroid subjects (24.1%) [60]. In addition, patients with subclinical hypothyroidism were more likely to have advanced colonic lesions compared with euthyroid subjects (Table 1) [60]. Boursi and colleagues conducted in the United Kingdom a case–control study including 20,990 CRC patients and 82,054 control subjects, aimed at evaluating the risk of CRC in patients with subclinical or manifest hypothyroidism but not taking hormonal therapy such as levothyroxine [61]. These patients had a modestly increased risk of developing cancer compared to individuals with normal thyroid function (OR = 1.16). At the same time, patients suffering from clinical or subclinical hypothyroidism but receiving levothyroxine presented a slightly reduced risk (OR = 0.92) compared to the same reference population. This protective effect was greater if the assumption of levothyroxine was continued for a long period, in that patients treated for more than 10 years showed a lower risk (OR = 0.68) than those receiving therapy for 5–10 years (OR = 0.88) [61]. The latter findings, however, could not be confirmed in a recent matched case–control study by Kuiper and colleagues, who analyzed 28,121 CRC patients and 106,086 controls in the Netherlands [62]. These authors found that levothyroxine treatment was not protective regardless of treatment duration and whatever the site of tumor onset, except for a borderline decrease in rectal cancer-related risk that was not statistically significant. Finally, a case–control study performed by L’Heureux and colleagues in Taiwan including 69,713 CRC patients and 69,713 controls reported that hypothyroidism was associated with a 22% lower risk of developing CRC, with an adjusted OR of 0.78 (Table 1) [63]. More precisely, they observed a 45% lower risk in the occurrence of rectal cancer with an OR of 0.55, while no changes in colon cancer prevalence were noticed. In addition, stratifying patients by age, a significant risk reduction was confirmed only for hypothyroid patients older than 50 years, with rectal cancer showing an OR of 0.54. In patients affected by HT, the risk of developing CRC was significantly reduced, with an OR of 0.63 [63]. The authors also adjusted the OR for medication and/or surgical treatment of thyroid disorders, and no statistically significant correlation emerged between previous treatment for hypothyroidism and the risk of CRC.

In conclusion, the available epidemiological data regarding the effects of hypothyroidism on CRC progression, summarized in Table 1, are still controversial. Whether these discordant findings are due to diverse ethnicity, diets, and/or lifestyles (e.g., smoking and alcohol consumption) of patients considered in the different case studies remains to be determined, and further well-controlled studies are required.

### 2.2. Hyperthyroidism, Graves’ Disease, and Colorectal Cancer

Various population studies were accomplished to assess the existence of a correlation between hyperthyroidism and susceptibility to the development of several types of neoplasms [65]. Hellevik and colleagues carried out in Norway a prospective study by measuring the TSH value in a population of 29,691 subjects who did not have a previous diagnosis of TD, and recorded the incidence of four neoplasms, namely colon, lung, breast, and prostate cancers, over a 9-year follow-up period (Table 1) [64]. By stratifying patients on the basis of TSH values, they demonstrated that those with plasma concentrations below the reference range (TSH < 0.50 mU/L) had the greatest risk of developing cancer in general. This evidence was consistent for lung and prostate cancer, with age- and sex-adjusted HR of 2.6 and 1.96, respectively. For colon cancer, it was possible to detect the same trend, with a higher incidence in patients with lower TSH (HR 1.42), but the small number of cases collected, and the consequent lack of statistical significance, did not allow for reliable conclusions to be drawn (Table 1) [64].

In the previously mentioned study of Boursi et al., an increased risk (OR = 1.21) of CRC in hyperthyroid patients was detected [61]. On the contrary, L’Heureux and colleagues found a 23% reduction in the risk of developing CRC (OR = 0.77), and 26% of developing colon cancer (OR = 0.74) [63]. If patients were stratified by age, the protective effect of hyperthyroidism was more pronounced in those with colon cancer under 50 (OR = 0.55) but did not reach statistical significance for patients with rectal cancer at any age (Table 1) [63].

Shu and colleagues recorded the incidence of neoplasms in 18,156 patients hospitalized for GD in Sweden and followed up for a mean period of 17 years [10]. They calculated the standardized incidence ratio (SIR) for each type of neoplasm with at least 20 identified cases. In this study, patients with GD had a significant decrease in the risk of colon cancer (SIR = 0.78) [10]. Similarly, Chen and colleagues compared the risk of developing cancer in a population of 5025 GD patients and 20,100 frequency matched non-GD patients in Taiwan [4]. GD patients had a significantly increased incidence rate for head/neck, hepatocellular, breast, prostate, and thyroid cancers, while they showed a significant risk reduction for colon, lung, bladder, uterus, cervix, and hematological neoplasms [4].

In conclusion, the effects of hyperthyroidism on CRC progression, summarized in Table 1, are still uncertain and require further studies to be elucidated.

### 2.3. The FT3/FT4 Ratio as Prognostic Factor in Metastatic Colorectal Cancer

Low T3 syndrome is a clinical condition characterized by low free T3 (FT3) levels associated with normal-to-low TSH and free T4 (FT4) values [66]. It is an endocrine disorder common in elderly patients, where a dysregulation of the hypothalamus–pituitary–thyroid axis could be the cause [67]. It is also frequently detected in patients who are critically ill or affected by acute pathologies, and in such cases, it is interpreted as an adaptation phenomenon capable of limiting energy consumption and the excessive catabolism observed in such pathological conditions [68,69,70]. In this context, several studies demonstrated the prognostic value of the low T3 syndrome, which is associated with a worse outcome [71,72]. It is plausible to hypothesize, given the absence of scientific evidence, that cancer can also determine the onset of this endocrine disorder, and that the reduction of peripheral T4 to T3 conversion may be a crucial event. The loss of muscle mass occurring in cachexia and the ensuing reduction in type 2 deiodinase (D2)-mediated deiodination, as well as the hepatic damage caused by tumor spreading with consequent impairment of +/type 1 deiodinase (D1)-mediated deiodination, could be considered as the pathological mechanisms arising in patients with metastatic CRC [73]. Since the plasma concentration ratio of FT3 and FT4 reflects the enzymatic activity of deiodinases, reduction in peripheral conversion of T4 to T3 results in lower T3 plasma concentration and hormonal deficiency at the tissue level. The relevance of the low T3 syndrome as prognostic factor in CRC was discovered by Pasqualetti and colleagues using data retrospectively extrapolated from a clinical trial (CORRECT trial). These authors noticed the best outcome in terms of overall survival (OS) and progression-free survival (PFS) in patients with metastatic chemorefractory CRC under Regorafenib therapy which had a higher FT3/FT4 ratio [73,74,75]. Among 760 patients with metastatic CRC belonging to the CORRECT study, 758 were selected based on the availability of FT3 and FT4 values: 503 were randomly assigned to regorafenib treatment and 255 to placebo. Each group was further divided by the tertiles of the FT3/FT4 ratio into low, intermediate, and high level. A direct proportionality between FT3/FT4 ratio, OS, and PFS was observed in both patients receiving the drug and those receiving the placebo [74,76]. Based on the correlation between alteration of peripheral deiodination and worse clinical outcome, the prognostic value of the FT3/FT4 ratio could be considered in the setup of prognostic scores aimed at assessing life expectancy in patients with metastatic CRC. The value of the low T3 syndrome in advanced cancer prognosis is not limited to CRC. In fact, it was identified as a strong prognostic factor for both overall survival and progression-free survival also in patients with metastatic renal cell carcinoma [77]. Similarly, a correlation between low T3 syndrome and unfavorable clinical outcome was reported for patients affected by hematological, lung, and brain tumors [78,79,80,81,82].

## 3. Thyroid Hormones, Thyroid Hormone Receptors, and Colorectal Cancer

THs affect target cells’ functions by either the classic genomic action, in which THs modulate transcription of responsive genes through interaction with specific nuclear TH receptors (THRs), or a number of non-genomic actions in which both T4 and T3 interact with receptors located in mitochondria, in the cytoplasm, or at the plasma membrane, eliciting rapid cellular responses [76,83]. At first, THs enter target cells by means of specific plasma membrane carriers, such as the monocarboxylate transporters MCT8 and MCT10, the organic anion transporters OATP1 and OATP3, and the L-type amino acid transporter LAT [84]. Inside the cells, T4 is then transformed into T3 by the deiodinases 1 (D1) or 2 (D2). The T3 binds THRs with 10- to 30-fold greater affinity compared to T4 [85]. Both THs can be inactivated by the deiodinase 3 (D3), which catalyzes the inner ring deiodination of T4 to reverse T3 (rT3), and of T3 to 3, 3’-diiodothyronine (T2). In the classic genomic mechanism of action, THRs act as ligand-dependent transcription factors by binding to specific DNA sequences present within the promoter regions of target genes, known as thyroid responsive elements (TREs), to modulate gene transcription [86,87]. THR proteins are encoded by the THRA gene (chromosome 17) via three different transcripts, THRα1, THRα2, and THRα3—of which only THRα1 is able to bind T_3_—and the THRB gene (chromosome 3) via the THRβ1 and THRβ2 transcripts [88,89,90,91,92]. Among non-genomic TH receptors, the plasma membrane αvβ3 integrin is the best known. It contains two TH binding sites, known as S1 and S2, of which S1 binds only T3 leading to intracellular activation of the PI3K, while S2 binds mainly T4 activating the intracellular MAPK cascade [93,94,95].

By acting through different receptors, THs are able to influence various cellular pathways involved in proliferation, migration, aerobic glycolysis, apoptosis, immune checkpoint, and angiogenesis. Thus, it is reasonable to presume that cancers are sensitive to changes of TH levels, both when they arise after tumor onset and before. However, the complex and multidirectional connections between THs and cancer do not allow for definitive conclusions to be drawn about THs as tumor promoters or suppressors. In fact, even if the prognostic significance of low TH levels in patients with established cancers, discussed in the previous paragraph, would suggest a protective role for THs in tumor progression, boosting effects of hyperthyroidism on several cancer types have been described, including breast, gastrointestinal, hematological, lung, prostate cancers, and melanoma [96].

The evidence that the biological activity of CRC is influenced by THs derives from the detection of both αvβ3 integrin and nuclear THRs in CRC cells, and from the study of the effects resulting from their activation [93,97]. THs were shown to augment the in vitro proliferation of a number of CRC-derived cell lines, such as the HCT-116, the FT-29, and the Colo205. Moreover, Iishi and colleagues reported the ability of T4 administration to increase the incidence of colon cancer induced in rats by azoxymethane [93,97]. Markowitz and colleagues first reported in 1989 the expression levels of THR transcripts in neoplastic colon and matched normal mucosa [98]. They found that THRβ1 mRNA was absent or strongly reduced in tumor tissues compared to normal matched tissues [98]. Later on, this observation was confirmed by Zhu and colleagues, which reported a reduction in CRC cells of THRβ1 mRNA and protein compared to normal colorectal mucosa, and an inverse correlation between gene expression and tumor size [99]. In vitro studies also showed that induction of the THRβ1 expression in cancer cells significantly inhibited their proliferation and ability to migrate, and that these effects were associated with a decrease in phosphorylation of protein kinase B (AKT) [100]. These observations are in agreement with a number of studies indicating that the reduced expression of THRs, due to THR genes hypermethylation, deletions, or somatic mutations, could cause unrestricted growth and dedifferentiation of tumor cells [100].

As above mentioned, the biological activity of THs is mainly determined by the intracellular levels of deiodinases that catalyze the production (D1 and D2) or degradation (D3) of T_3_ [101]. In several human cancers, a dysregulation of deiodinases expression is frequently encountered, and affects the intracellular TH actions [102,103,104,105]. In particular, D3 is the one most commonly connected to human cancers [104,105]. It is defined an oncofetal protein, highly expressed in cells during embryonic development, while during adult life it is found in physiological conditions only in the central nervous system and skin, and is often re-expressed at high levels in malignant cell lines and solid tumors, including colon carcinoma [100,104,105]. Of note, D2 and D3 are modulated in opposite ways by the WNT/β-Catenin pathway, the expression of D2 being down-regulated and that of D3 up-regulated at the same time [30,31,32,33,34,35,106]. The overexpression of D3 has been demonstrated in vitro as well as in vivo by immunohistochemical analysis of colon cancer tissue samples, where the level of D3 protein was greater in adenomas and colon carcinomas compared to normal tissues [106]. These findings are consistent with the observation by Rose and Davis of increased rT_3_ plasma levels in 24% of patients with metastatic colon cancer [107]. In this context, of particular interest are the results of Catalano and colleagues, who demonstrated that intracellular T3 is effective in inducing differentiation, growth arrest, and chemosensitization of CRC stem cells, thus preventing their expansion [102,108].

**Figure 1:**
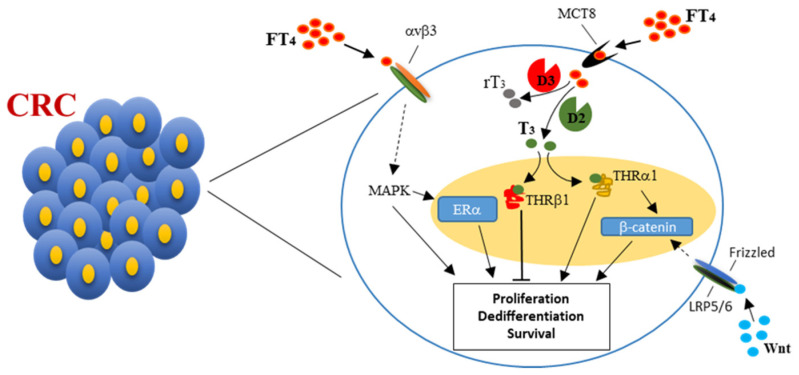
**Schematic representation of thyroid hormones action on colorectal cancer (CRC) cells**. FT4, free-thyroxine; αvβ3, integrin receptor; MCT8, monocarboxylate transporter 8; D2, type 2 deiodinase; D3, type 3 deiodinase; MAPK, mitogen-activated protein kinases; ER, estrogen receptor; THR, thyroid hormone receptor. See text for explanation.

All together, the above findings seem to suggest that a condition of intracellular hypothyroidism due to either a reduced expression and/or function of THRβ1 or decreased T_3_ availability could promote CRC progression (Figure 1).

On the other hand, based on the evidence that TRα1 controls intestinal development and homeostasis processes through the WNT pathway, Kress and colleagues investigated its onco-inducing properties related to WNT/β-Catenin pathway activation [30,31,32,33,34,35]. Studies carried out on animal models demonstrated that the overexpression of TRα1 led to hyperproliferation of the mucosa and development of adenomas in small and large intestine. Moreover, the overexpression of TRα1 was associated with that of WNT pathway components (i.e., β-Catenin, c-Myc, and Cyclin D1), which suggests the existence of a cooperation between TRα1 and WNT pathways in the tumorigenic process [31,32] (Figure 1).

Yang and colleagues examined the role of the plasma membrane αvβ3 receptor in CRC cells and found that binding of T4 to the αvβ3 integrin induces the expression of genes involved in CRC cell proliferation [109]. The importance of this pathway is confirmed by the effects of the tetraiodothyroacetic acid (TETRAC), a deaminated analogue of T4, and of its nano-derivative (Nanotetrac) obtained in vitro and in experimental animal models [110,111]. TETRAC and Nanotetrac compete with T4 for the binding site on the integrin αvβ3, which results in reduced cell proliferation and angiogenesis, and apoptosis induction [110,111,112]. This is well in agreement with the reported ability of the αvβ3 receptor to activate the MAPK pathway, and to induce neoangiogenesis following TH binding [98] (Figure 1). In this context, it is worth considering that T4 activates the αvβ3 integrin at physiological free-hormone concentrations, while supraphysiological free-T3 serum levels are needed to prompt integrin-dependent cell proliferation [109]. Remarkably, in a clinical study involving patients with advanced solid cancers, Hercbergs and colleagues demonstrated that treatment with methimazole and T3 to prevent hypothyroidism (euthyroid hypothyroxinemia) had a significant positive impact on patient’s survival [113]. These findings appear to corroborate the data described above, indicating a direct proportionality between FT3/FT4 ratio, OS, and PFS in metastatic CRC patients [74,75].

## 4. Thyroid Hormones and Estrogens Cross-Talk in Colorectal Cancer

Besides being associated to cancers of reproductive organs, estrogens have also been suspected to play a role in the progression of different human neoplasms including lung and gastrointestinal cancers [114,115]. Males and females differ in CRC site presentation, with female patients commonly having malignant lesions in the proximal colon, while males more frequently in the distal colon [116]. Moreover, CRC incidence has been shown to be higher in males that in females, and higher in females above 65 than in younger ones [117,118,119]. This evidence suggests a possible protective role of estrogens in CRC progression [113]. Estrogens affect cellular functions by binding to two nuclear receptors: the estrogen receptors alpha (ERα) and beta (ERβ) [120,121]. Upon ligand binding, these receptors modulate gene transcription by interacting with specific DNA sequences (estrogen-responsive element, ERE) located in the promoter region of their target genes. ERs can form complexes with other transcription factors, including Sp1, Ap1, and NF-kB. In addition, as for THs, estrogens may affect cell functions through binding to receptors located either on the plasma membrane or on the nuclear membrane. The two ERs were reported to have opposite effects on CRC cells, with ERα promoting colon cancer cell proliferation, and ERβ causing apoptosis of colon malignant cells [120,121]. Specifically, estrogen binding to ERα stimulates the ERK/MAPK and PI3K/AKT pathways leading to an increased cellular proliferation, while ERβ activation triggers anti-proliferative signals through the p38/MAPK pathway and caspase 3 activation [119,120]. Although ERβ is the predominant estrogen receptor in normal colonic epithelium, its expression is frequently reduced or lost during CRC progression, suggesting that estrogen signaling may play a role in disease evolution [120,121].

A number of experimental results indicated that THs could affect estrogen-dependent effects on CRC cells in several ways [122,123,124,125]. In fact, THs have been shown to increase the expression of ERs, and T4, through the αvβ3 integrin receptor, may activate the MAPK-mediated phosphorylation of the nuclear ERα [122,123,124] (Figure 1). This phosphorylation affects ER ability to interact with chromatin, to recruit coregulators, and to modulate gene expression even in the absence of estrogen [124]. In addition, TREs and ERs response element (ERE) share an identical half-site, and THRs can bind to the ERE, thus potentially influencing transcription of estrogen responsive genes [125].

## 5. Conclusions

Although epidemiological and clinical experimental data so far available have produced conflicting results on the ability of THs to affect CRC progression, information obtained at the molecular level on the imbalance in THR expression and signaling, and on TH cross-talk with the WNT/β-Catenin pathway and estrogens seem to suggest a role of THs in prompting CRC development. These observations should warrant further clinical and molecular investigations aimed at better defining the potential involvement of THs in CRC. This knowledge could lead to an improved clinical management of CRC patients, exploiting the potential therapeutic value of TH levels modulation in a tumor that still represents the second deadliest cancer worldwide.

## Figures and Tables

**Table 1:** **Association of thyroid diseases with colorectal cancer**. CI, confidence interval; CRC, colorectal cancer; OR, odds ratio; HR, hazard ratio; SIR, standardized incidence ratio; IRR, incidence rate ratio.

First Author (Ref.)	Year	Country	Thyroid Disease	Cancer	Patients/ Control	Risk (95% CI)	*p*
Rennert et al. [58]	2010	Israel	Hypothyroidism on levothyroxine	CRC	2566/2566	OR 0.59 (0.43–0.82)	0.001
Friedman et al. [59]	2011	California	Hypothyroidism on levothyroxine	Rectum Colon	4729/235,925 12,207/608,296	OR Male 0.66 (0.45–0.97) OR Female 0.97 (0.78–1.19)	0.03 0.74 0.18 0.06
						OR Male 0.87 (0.71–1.07) OR Female 0.90 (0.81–1.07)	
Chen et al. [5]	2013	Taiwan	Hashimoto	CRC	1521/6084	HR 4.76 (1.36–16.6)	<0.05
Mu et al. [60]	2015	China	Subclinical Hypothyroidism	CRC	273/819	OR 1.689 (1.207–2.362)	0.002
Boursi et al. [61]	2015	United Kingdom	Hypothyroidism	CRC	20,990/82,504	OR 1.16 (1.08–1.24)	<0.001
			Hypothyroidism on levothyroxine			OR 0.92 (0.86–0.98)	0.009
L’Heureux et al. [63]	2019	Taiwan	Hypothyroidism	CRC	69,713/69,713	0.78 (0.65–0.94	<0.001
Kuiper et al. [62]	2022	Netherlands	Hypothyroidism	CRC	28,121/106,086	OR 0.95 (0.88–1.01)	>0.05
Hellevik et al. [64]	2009	Norway	Hyperthyroidism	Colon	29,691	HR 1.42	>0.05
Boursi et al. [61]	2015	United Kingdom	Hyperthyroidism	CRC	20,990/82,504	OR 1.21 (1.08–1.36)	0.001
L’Heureux et al. [63]	2019	Taiwan	Hyperthyroidism	CRC	69,713/69,713	OR 0.77 (0.69–0.86)	<0.001
Shu et al. [10]	2010	Sweden	Graves’ disease	Colon	18,156	SIR 0.78 (0.61–0.97)	<0.05
Chen et al. [4]	2013	Taiwan	Graves’ disease	Colon	5025/20,100	IRR 0.61 (0.53–0.69)	<0.001

## Data Availability

Not applicable.
